# Complement activation in IgA nephropathy

**DOI:** 10.1007/s00281-021-00882-9

**Published:** 2021-08-11

**Authors:** Nicholas R. Medjeral-Thomas, H. Terence Cook, Matthew C. Pickering

**Affiliations:** Centre for Inflammatory Disease, Department of Immunology and Inflammation, Imperial College London, London, W12 0NN UK

**Keywords:** Complement, IgA nephropathy, Immunology, Pathology

## Abstract

IgA nephropathy pathogenesis is incompletely understood, and this limits the development of disease-specific biomarkers and effective therapies. Evidence of complement activity in IgA nephropathy is well established. However, a growing body of research indicates complement activity is an important contributor to IgA nephropathy pathology. In particular, multiple associations have been identified between complement alternative, lectin and terminal pathway proteins and IgA nephropathy severity. Recently, we have also gained insight into possible mechanisms that could link glomerular IgA deposition, complement activity, glomerular inflammation and disease severity. Ongoing clinical trials of therapeutic complement inhibitors will provide insight into the importance of complement activity to IgA nephropathy pathogenesis. Further research into mechanisms of complement activity is essential to improving our understanding and management of patients with IgA nephropathy.

The pathogenesis of IgA nephropathy (IgAN) is incompletely understood [1]. Consequently, the availability of effective treatments and accurate biomarkers of disease severity is limited. IgAN is the most common primary glomerular disease globally and often affects young adults [2], with significant personal and socioeconomic damage. Therefore, improved understanding of IgAN pathogenesis is of urgent importance. Evidence of glomerular complement (C)3 deposition is common in IgAN [3], but the pathogenic relevance of complement activity to IgAN is unclear. A growing body of evidence suggests that activation of the lectin pathway (LP), alternative pathway (AP) and terminal pathway (TP) of complement are pathogenic in IgAN. Also, recent research has demonstrated the importance of effective complement regulation in IgAN. These developments have been paralleled by the increasing availability of therapeutic agents that inhibit complement pathway activation [4]. Consequently, there is an imminent need to appropriately and safely select patients for therapeutic inhibition of specific complement pathways. Understanding which therapies are most likely to provide benefit and who to treat will depend on insight into the mechanisms and impact of complement activity in IgAN. We will review evidence of complement activity in IgAN, discuss possible pathogenic mechanisms for complement in IgAN and highlight areas requiring further research.

## Current theories of IgA nephropathy pathogenesis

The multihit theory of IgAN pathogenesis is based on an extensive body of evidence, reviewed in other articles in this issue of *Seminars in Immunopathology*. However, a number of clinical, pathological and epidemiological observations are not completely explained by this theory. In particular, IgAN encompasses a wide spectrum of clinical phenotypes, histology patterns and severity outcomes. Up to 16% of healthy individuals have deposited glomerular IgA with no signs of kidney disease [5]. Approximately 5% of patients with biopsy-proven IgAN will have mild or no impairment of kidney function, and minor histology glomerular changes. However, for 40% of patients, kidney function will deteriorate and progress to kidney failure over 30 years [6] and a small proportion of patients will present with rapidly progressive glomerulonephritis and glomerular crescents [7]. The prevalence of IgAN varies widely between countries, with the highest prevalence in Singapore and Japan [8]. The clinical severity of IgAN is also associated with ethnicity. In Canada, individuals of Pacific Asian origin have increased risk of kidney failure from IgAN compared with individuals of European ancestry [9]. It is unclear how de-galactosylation of IgA1, subsequent autoantibody production and glomerular deposition of immune complexes, which form the basis of the multihit theory of IgAN pathogenesis, can account for this spectrum of disease severity. It seems likely that the pathogenesis of each subtype of IgAN is different. Autoimmune and pro-inflammatory pathways could influence each subtype to a different extent.

## The complement system

**Fig. 1:**
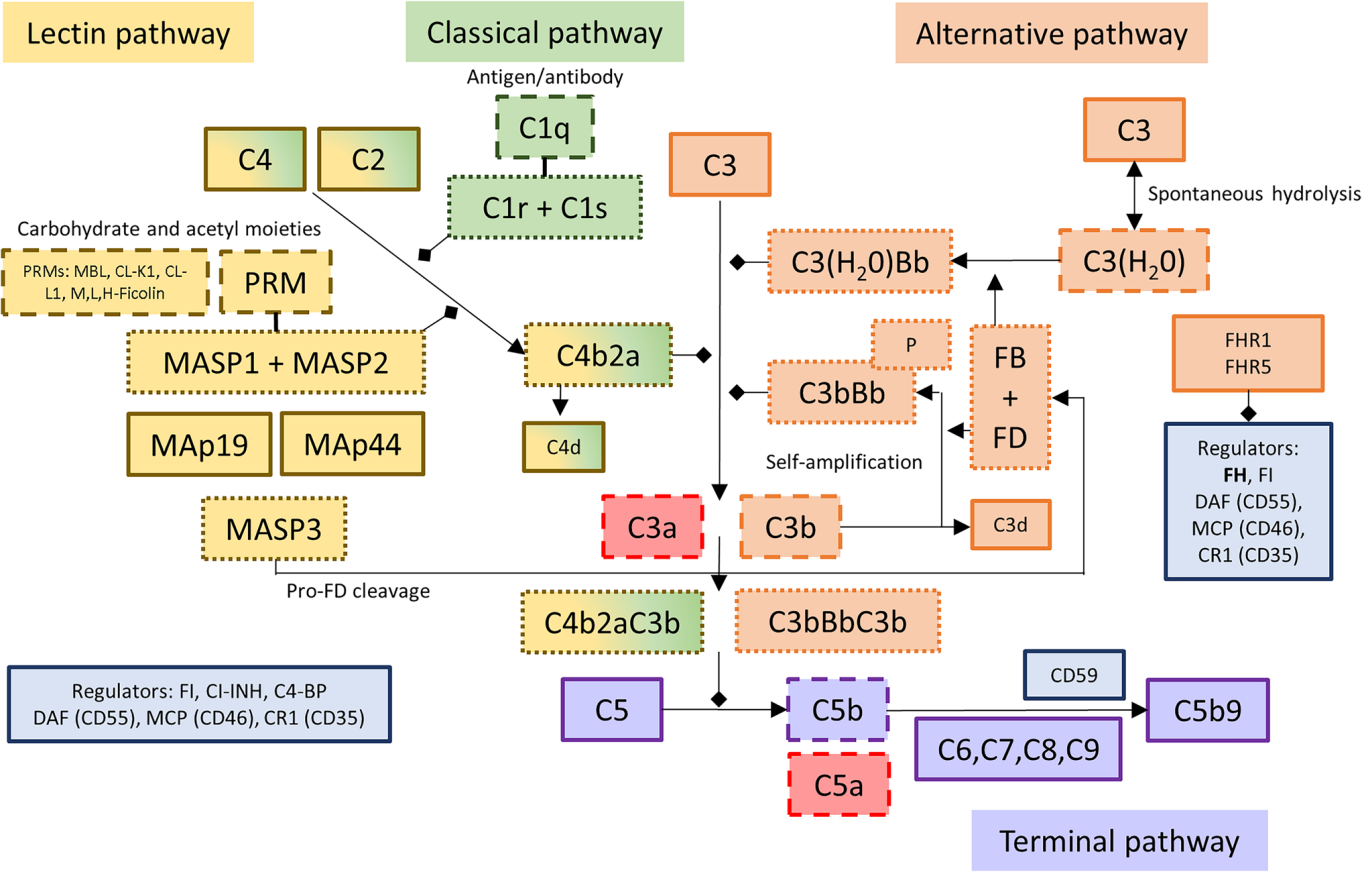
Complement system. The complement system is activated through the classical, lectin or alternative pathway. The classical and lectin pathways are initiated by the recognition of non-host or damaged cell surfaces. The alternative pathway is constitutively and spontaneously activated by the hydrolysis of the C3 to expose the thioester domain and form C3(H_2_O). After factor B (FB) has been activated to Bb by factor D (FD), Bb interacts with C3(H_2_O) to form a C3 convertase (C3(H_2_O)Bb). C3 convertases cleave C3 into the anaphylatoxin C3a and C3b. Through activation of C4 and C2 by the C1 complex or lectin pathway PRM-MASP complex, the classical and lectin pathways form the C3 convertase C4b2a. C3b deposited on surfaces can form additional C3 convertases, C3bBb, after binding activated FB, and this drives the C3 amplification loop. Binding of additional C3b molecules to existing C3 convertases leads to the formation of C5 convertases (C4b2aC3b, C3bBbC3b) that cleave C5 into C5a, a potent anaphylatoxin, and C5b. C5b binds to C6, C7, C8 and C9 and forms the membrane attack complex (C5b9), which causes target cell damage. Complement activation is tightly controlled by fluid-phase and membrane-bound regulators. Factor H (FH) is the major negative regulator of the alternative pathway and controls C3 activation. Other soluble regulatory proteins include C1-INH, C4-BP and factor I (FI). Nearly all cells express membrane-bound regulators including DAF (CD55), MCP (CD46, not present on erythrocytes), CR1 (CD35) and CD59. Properdin (P) stabilises the AP C3 convertase and is a positive regulator of the complement system. The factor H–related proteins 1 (FHR1) and 5 (FHR5) may impair complement activation by competing for C3b binding with FH

The complement system is a network of activating and regulating proteins (Fig. 1) that mark damaged and non-self cells and tissue [10]. Complement activation self-amplifies and leads to inflammation. Effective but appropriately regulated complement activity is essential to innate and adaptive immunity and homeostasis [11]. The complement system is characterised by multiple variants to protein structure, abundance and activation, the prevalence of which is often associated with ethnicity. Clinical observations indicate that variants in AP and LP activity could provide the pathogenic link between glomerular IgA deposition and glomerular inflammation and injury. A rare subtype of familial C3 glomerulopathy caused by AP dysregulation, CFHR5 nephropathy, shares many clinical and histological features with IgAN [12, 13]. The LP is essential to innate immunity at mucosal surfaces, and IgAN is often characterised by flares coincidental with respiratory and gastrointestinal inflammation [1].

Complement activation can be triggered by the classical pathway (CP), LP or AP (Fig. 1). The CP is activated by binding of the pattern recognition molecule (PRM) C1q to pathogen-associated molecular patterns (PAMPs), damage-associated molecular patterns (DAMPs) or IgM- and IgG-containing immune complexes, or via molecules such as CRP [14]. Classical pathway activity can be detected by the identification of C1q by tissue immunostaining. The absence of C1q in the vast majority of IgAN kidney biopsies suggests the CP does not contribute significantly to IgAN pathogenesis [15].

The LP is triggered by the interaction of PRM with carbohydrate PAMPs and DAMPs located, for example, on non-self cells [16]. The detection of C4d, a cleaved fragment of C4 activation, in the absence of C1q is immunohistological evidence of LP activation. The LP PRMs include mannan-binding lectin (MBL), M-ficolin (Ficolin-1), L-ficolin (Ficolin-2), H-ficolin (Ficolin-3), collectin liver 1 (CL-L1, also referred to as collectin-10, CL-10) and collectin kidney (CL-K1, also referred to as collectin-11, CL-11) [16]. The PRMs circulate in complex with proenzyme dimers. PRM binding activates the proenzymes to serine proteases. The LP proteases are named MBL-associated serine protease (MASP)-1, MASP-2 and MASP-3 [17]. Two non-proteolytic proteins can also be found in LP PRM complexes: MBL-associated protein (MAp)44 and MAp19. Each MASP and MAp can affect LP activation differently. MASP-2 can cleave and activate C4 and C2 to form a C3 convertase (C4bC2a) [17]. MASP-1 activates MASP-2 and can cleave C2 but not C4 and is therefore unable to form a C3 convertase in the absence of MASP-2. MASP-3 does not cleave either C4 or C2. Recent data demonstrate MASP-3 is the protease responsible for pro-factor D activation. Factor D is a key component of AP activation [18]. MASP-1, MASP-3 and MAp44 are alternative splice products of the *MASP1* gene, and MASP-2 and MAp19 are alternative splice products of the *MASP2* gene [19]. Therefore, genetic variants can influence the balance of circulating proteins that, despite close structural and amino acid sequence homology, have different effects on the lectin and alternative complement pathways.

Alternative pathway activation occurs via constant tick-over hydrolysis of C3, exposing a thioester domain binding site (Fig. 1). A C3 convertase is formed from the binding of hydrolysed C3 to activated factor B (Bb) [14]. Bb is a serine esterase produced from the activation of factor B by factor D. The C3 convertase cleaves more C3 to the activated C3b. The C3b thioester domain binds neighbouring surfaces through covalent attachment to hydroxyl and amine groups on carbohydrates and proteins. C3b then binds more Bb to form the AP C3 convertase, C3bBb. C3 cleavage releases the anaphylatoxin C3a. Properdin can bind and stabilise the C3 convertase. In the absence of AP regulation, multiple C3 molecules are cleaved and activated, with further C3 convertase formation and complement activity amplification. As the density of activated C3b increases, C3 convertases bind C3b to form C5 convertases [14]. Consequently, AP-dependent complement amplification cascades to TP activity. The C5 convertase cleaves C5 to the anaphylatoxin C5a and fragment C5b. C5b binds C6–C9 to from C5b9, the membrane attack complex. The membrane attack complex is a pore-like structure that inserts in cell membranes and promotes lysis of non-nucleated cells and gram-negative bacteria and injurious pathways in nucleated cells [11].

Effective complement system regulation is essential to target inflammation and prevent host cell injury. In addition to physical characteristics that limit complement activation, the spontaneous decay of the C3 and C5 convertase [20], a number of surface-bound and fluid-phase complement pathway regulators limit complement activity and cascade progression. CD59, CD35 (CR1) and CD55 are important surface-bound complement regulators. The LP and CP are also regulated by C4-binding protein, factor I and C1 inhibitor, a serpin-type inhibitor of C1r, C1s, MASP-1 and MASP-2 [16]. Factor H (FH) and factor I are also essential regulators of the AP [21]. Factor I is a protease that, in the presence of cofactor molecules, cleaves C3b to iC3b. iC3b is unable to participate further in complement activation and is rapidly cleaved to C3c and the surface-bound C3dg. Complement FH is an abundant plasma protein that regulates the alternative pathway in the fluid-phase and on cellular surfaces. FH prevents binding of Bb to C3b, accelerates decay of the AP C3 convertase and has co-factor activity for FI-mediated proteolytic inactivation of C3b [21]. Recently, the potential for the factor H–related (FHR) proteins to interfere with complement regulation has been demonstrated. The FHR proteins show high sequence identity with FH, especially at the surface and C3b binding domains. However, the FHR proteins lack the complement-regulating domains of FH. This results in potential competition by FHR proteins for complement binding and subsequent impairment of FH complement regulation [22]. The importance of FH is evidenced by diseases caused by its impaired function. Mutations to and autoantibodies against the FH surface-recognition domains are associated with atypical haemolytic uraemic syndrome (aHUS). Factor H deficiency, or the attenuated binding of FH to C3b by mutant FHR proteins, leads to C3 glomerulopathy [12].

Complement activity can be detected with immunostaining evidence of C3 deposition. In kidney biopsy immunostaining for C3, the antibodies typically used are those that are raised against C3c. These antibodies will also react with C3b and iC3b. However, C3dg usually needs to be detected with an anti-C3d antibody. Complement TP activity can be detected by the tissue deposition of C9 or C5b9. Although tissue C3, C9 and C5b-9 are clear evidence of complement activation, staining for specific C3 proteins can provide information on the timing of pathway activation. For example, whilst C3c staining resolves within 24 h after the cessation of complement activity C3dg remains detectable for weeks. So measuring the presence of both C3 fragments can provide information as to whether or not complement activation is ongoing (C3c and C3dg) or not (C3dg only). C5b9 can still be detected many months after activation [23, 24].

## Evidence of complement activation in IgA nephropathy

### Histology

Immunohistochemical evidence of activated C3 fragments was noted in the first description of IgAN by Berger in 1968 [25]. At the time, the identification of glomerular complement proteins, referred to as β1C-globulin, in the absence of systemic autoimmune or infectious disease was controversial. However, the finding was replicated in a number of other IgAN biopsy series [15], which confirmed evidence of local, glomerular C3 activation in IgAN. More recently, immunostaining and proteomic studies have provided detailed evidence of specific complement protein glomerular deposition in IgAN and identified a number of associations with IgAN severity.

IgAN biopsy series have established that glomerular IgA and C3 are often accompanied by properdin, C3dg and C5b9 [3, 25]. The presence of these proteins is evidence of AP and TP activity. Glomerular C4d and absent C1q can be detected in roughly 40% of patients and is indicative of LP activity. This is further supported by the identification of MBL, L-ficolin and MASP2 and MASP1/3 (the antibody used did not differentiate the two proteases) in about 25% of IgAN cases in series from Japan and the Netherlands, and the co-deposition of MBL/MASP-1 with glomerular AP and TP proteins [26, 27].

**Fig. 2:**
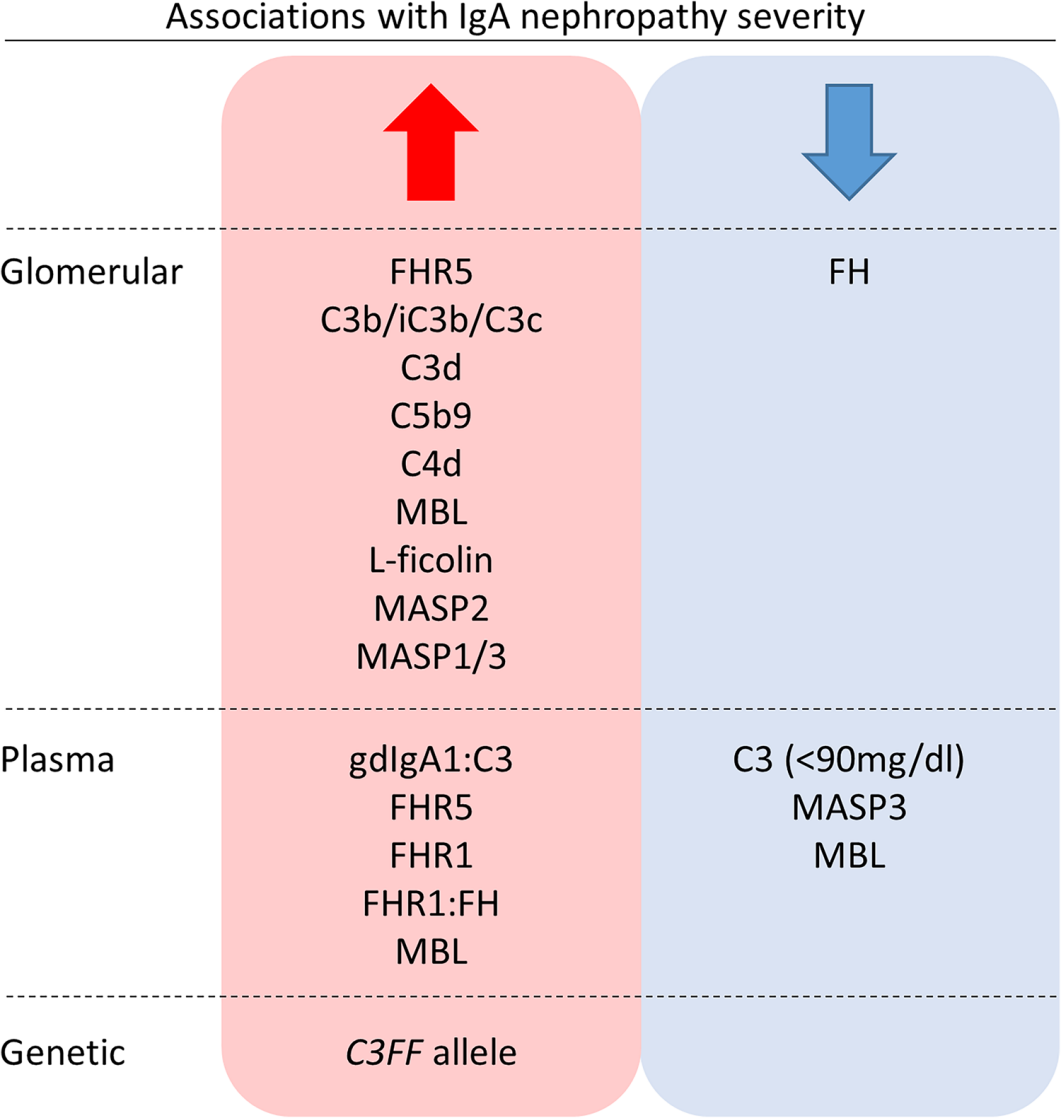
Associations between complement proteins and polymorphisms and IgA nephropathy severity. Schematic summary of associations identified between complement proteins and polymorphisms and the severity of IgA nephropathy. Glomerular changes refer to associations identified from immunostaining analyses of pathology series, plasma changes refer to serology evidence of complement protein levels and genetic associations refer to the association between polymorphism alleles and IgAN disease severity. See main text for further details and references

Glomerular LP and AP protein deposition are also associated with IgAN severity (Fig. 2). Multivariate analysis of a cohort of 283 IgAN patients from Spain identified associations between glomerular C4d deposition and the development of end-stage kidney disease (ESKD) [28]. Roos et al. demonstrated that glomerular deposition of MBL, L-ficolin, MASP2, MASP1/3 and C4d was associated with proteinuria, raised serum creatinine and future kidney failure [27]. Recent analysis of an IgAN cohort with preserved glomerular filtration rates from Spain demonstrated associations between biopsy evidence of C4d deposition, increased proteinuria at biopsy, increased proteinuria during follow-up, disease flares, more rapid loss of the estimated glomerular filtration rate (eGFR) and a higher rate of kidney failure [29]. This suggests LP activation may influence IgAN severity at early stages of disease pathogenesis and natural history, when renal function remains relatively preserved and before the establishment of significant kidney scarring. Finally, a recent study of 128 IgAN and IgA vasculitis biopsies from the Netherlands found that patients with evidence of both C4d and microangiopathy on diagnostic biopsy had reduced renal survival [30].

The abundance of mesangial and capillary wall C3 deposition, which can be quantified from immunofluorescence studies, correlates with IgAN severity and progression. This is supported by the finding that glomerular C3 is associated with morphological features of glomerular inflammation and injury, such as mesangial hypercellularity, segmental sclerosis and the presence of cellular crescents, both of which suggest AP activity contributes to glomerular injury [31].

**Fig. 3:**
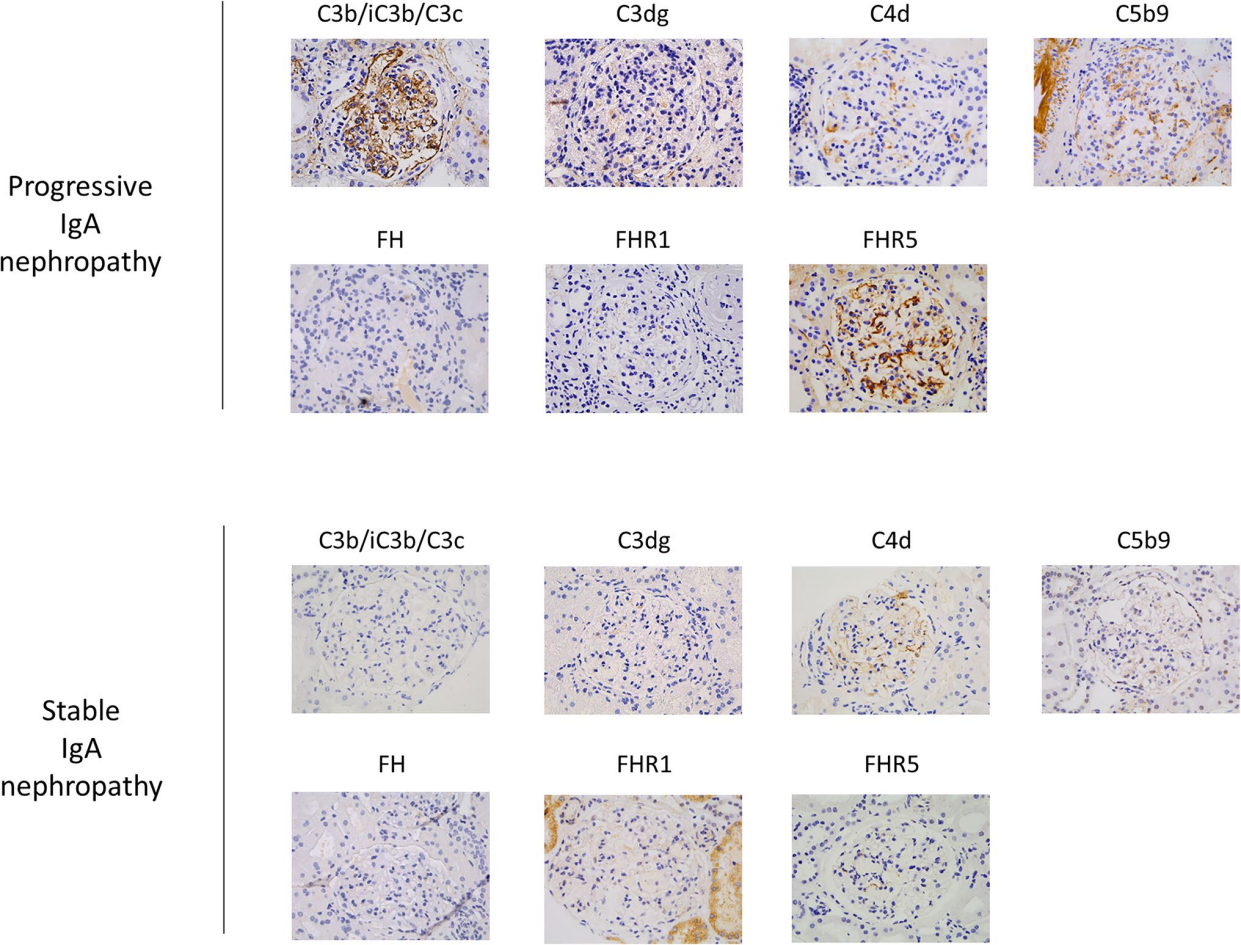
Representative images of kidney biopsy immunohistochemistry staining for complement pathway proteins in IgA nephropathy. Representative images from the diagnostic biopsy of one patient with progressive IgA nephropathy (IgAN) and one patient with stable IgAN. Progressive IgAN was defined as the occurrence of 40% loss of estimated glomerular filtration rate or end-stage kidney disease over 5 years from diagnosis. Images show immunohistochemistry staining using antibodies to complement (C)3b/iC3b/C3c, C3dg, C4d, C5b9 (the membrane attack complex), factor H (FH), factor H–related protein (FHR)1 and FHR5. All images are at 400 × magnification. Patients were enrolled in the Causes and Predictors of Outcome in IgA Nephropathy study (UK National Research Ethics Service Committee number 14/LO/0155), and the images are taken from research reported in [34]

Recently, proteins that can impair AP regulation have been identified in IgAN biopsy tissue. Factor H–related protein 5 (FHR5) attenuates the regulation of AP activation and amplification by FH [32]. Glomerular FHR5 was identified in similar mesangial distribution to IgA and C3 in all IgAN cases from a biopsy series of glomerular diseases from Australia [33]. In an IgAN patient cohort from the UK, glomerular FHR5 abundance correlated with the magnitude of glomerular C3b/iC3b/C3c, C3dg and C5b9 deposition and showed negative associations with glomerular FH [34]. Furthermore, glomerular deposition of FHR5, C3, C3dg, C4d and C5b9 was associated with progressive IgAN severity [34] (Fig. 3). Associations between glomerular FHR5 deposition and histology biomarkers of IgAN severity have been replicated in a cohort of 56 IgAN patients from China [35]. Patients with endocapillary hypercellularity and segmental sclerosis had more intense glomerular FHR5 deposition, and the abundance of glomerular FHR5 correlated with glomerular C3. Also, FHR5 co-localised with IgA and with C3c in glomerular mesangial and capillary areas [35]. Interestingly, kidney immunohistochemistry (IHC) for factor H–related protein 1 (FHR1), which is also predicted to interfere with FH-dependent AP regulation and is linked to the risk of IgAN in genome-wide association studies (see below), did not correlate with IgAN severity [34].

Importantly, the presence of complement proteins in glomeruli has been confirmed by proteomic analysis of micro-dissected glomerular cross-sections from 25 IgAN patients from Norway, 9 of whom progressed to ESKD [36]. Progressive IgAN was associated with the glomerular abundance of TP proteins, C4, C4-binding protein and FHR5 [36]. In addition to demonstrating the complexity of glomerular protein deposition in IgAN, these data validate associations identified by IHC. Together these results suggest complement AP dysregulation by FHR5, and LP and TP activity contribute to the pathogenesis of severe, progressive IgAN.

### Serology

Although hypocomplementaemia, as defined by clinical parameters of low serum C3 and C4, is rare in IgAN, other serology markers of complement activity can be detected and are associated with disease severity in IgAN. Analysis of 343 IgAN patients from Korea demonstrated serum C3 levels of less than 90 mg/dl at diagnosis associated with increased risk of doubling of serum creatinine over 54 months and significantly lower 10-year renal survival [31]. Reduced plasma C3 was also associated with increased mesangial C3 deposition, which was itself an independent predictor of adverse renal events. These data demonstrated links between serology evidence of complement activity and tissue-bound complement activation in IgAN [31]. Supportive of these findings, increased levels of plasma C3 activation products have been identified in IgAN patients [37] and are associated with proteinuria, haematuria and subsequent deterioration in renal function [38]. Furthermore, the ratio of plasma galactose-deficient IgA1 and C3 was independently associated with progression to chronic kidney disease or kidney failure in a large cohort of IgAN patients from China [39]. This indicates that the combination of higher circulating galactose-deficient IgA1 and lower serum C3, which results from an increased AP complement activity, contributes to kidney injury in IgAN.

Serology evidence of imbalances of AP-regulating proteins has been observed in IgAN (Fig. 2). Two large IgAN cohort studies, one from China and one from the UK, identified higher plasma FHR5 in IgAN patients than in healthy controls [40, 41]. In the UK cohort, higher FHR5 levels were associated with histology features of severe disease [40]. In the Chinese cohort, FHR5 levels correlated with proteinuria, hypertension and reduced eGFR [41]. Two independent studies of IgAN patients from Europe demonstrated significantly higher plasma FHR1 concentrations in IgAN compared to healthy controls and cohorts with polycystic kidney disease [40, 42]. Higher FHR1 was associated with reduced eGFR and more severe, progressive IgAN. Although plasma levels of the key AP regulator, FH, were not associated with disease severity, the ratio of FHR1/FH, an indicator of the relative abundance of dysregulating and regulating proteins, was associated with progressive disease [40, 42]. These data indicate that elevated circulating FHR5 and FHR1 may contribute to IgAN pathogenesis by impairing FH-dependent AP regulation and influencing the severity of glomerular inflammation and injury.

Multiple associations have been identified between circulating LP protein levels and IgAN (Fig. 2). However, the relationship between LP proteins and IgAN severity is complex. This may reflect the different impact of each LP protein on complement activity. MBL deficiency, defined as plasma levels less than 100 ng/ml, was associated with 50% loss of eGFR or ESKD in IgAN [43]. This was unexpected given aforementioned associations between markers of LP activity and IgAN severity. However, more cases of prodromal infection and gross haematuria were identified in MBL-deficient patients [43]. Therefore, MBL deficiency may contribute to infection susceptibility, IgAN exacerbation frequency and disease severity. Conversely, high MBL levels (greater than 3540 ng/ml) were also associated with markers of IgAN severity including increased proteinuria and the presence of cellular crescents, although associations were lost after multivariate adjustment [43]. This indicates that high plasma MBL could predispose to inappropriate lectin pathway activity, increased glomerular inflammation and IgAN severity. The apparently contradictory associations between IgAN severity and both high and low plasma MBL could be explained by different pathogenic mechanisms contributing to disease progression in different IgAN sub-cohorts.

Quantification of plasma concentrations of all LP proteins in a cohort of 323 patients from the UK identified associations between increased M-ficolin, L-ficolin, MASP-1 and MAp19 levels in IgAN patients compared to healthy controls [34]. Plasma MASP-3 levels were lower in IgAN. Reduced MASP-3 was associated with clinical and histology markers of IgAN severity [34]. It is unclear whether associations between low MASP-3 and IgAN severity are secondary to fluid-phase consumption or tissue deposition of MASP-3 and, given the aforementioned evidence that MASP-3 is responsible for factor D activation, whether these findings reflect increased AP or LP activity.

### Genetic

Large, multinational genetic studies have identified AP and LP variants that are associated with the risk of developing IgAN. Genome-wide association studies that included different ethnicity cohorts identified an allele within the FH gene that is associated with protection from IgAN. The protective allele tags a deletion polymorphism of the FHR1 and FHR3 genes (del*CFHR3-R1*) [44]. The presence of this allele reduces the risk of developing IgAN by 26% in heterozygosity (odds ratio (OR) = 0.74) and 45% in homozygosity (OR = 0.55) [45]. In an independent IgAN cohort from China, del*CFHR3-R1* was associated with reduced mesangial C3 deposition and tubulointerstitial injury, suggestive of reduced complement dependent kidney injury [46]. del*CFHR3-R1* was associated with higher circulating FH and intact C3 levels, lower plasma C3a levels and less mesangial C3 deposition [46]. The protection from IgAN associated with del*CFHR3-R1* could be explained by reduced competition for FH, enhanced complement AP regulation and subsequent limited inflammation. Additionally, four FH genetic variants predicted to impair FH function or expression were identified in a Spanish cohort of 106 IgAN patients [42]. This suggests impaired AP regulation secondary to quantitative or qualitative FH deficiency predisposes individuals to IgAN.

As mentioned, FHR5 can deregulate complement activity through competition with FH. Genetic mutations that increase the hetero-dimerisation of FHR5, and thereby increase the ability to compete with FH ligand [32], are strongly associated with a familial C3 glomerulopathy referred to as CFHR5 nephropathy [13]. CFHR5 nephropathy is phenotypically similar to IgAN. Additionally, rare FHR5 gene variants predicted to increase FHR5 binding capacity to C3b, again attenuating FH regulation, have been identified in IgAN [47]. These results are further evidence that impaired FH-dependent complement regulation by FHR5 can contribute to complement-mediated kidney injury in IgAN.

Additional to FH deregulation, genetic variants of C3 allele R102B are also associated with IgAN. The three polymorphic variants at this allele, homozygote *C3SS* (slow), homozygote *C3FF* (fast) and heterozygote *C3FS* are differentiated by the replacement of arginine amino acid in *C3S* with glycine amine acid in the *C3F* (the nomenclature refers to the speed or protein gel electrophoresis travel). Analysis of a large chronic kidney disease (CKD) cohort from the UK demonstrated associations between the *C3F* allele and markers of IgAN severity [48]. The *C3F* allele was associated with rapid progression of eGFR loss, defined as loss of at least 3 ml/min/1.73 m^2^ per year; glomerular C3 deposition; and progression of CKD in patients with IgAN, but not those with other glomerulopathies [48]. These data could reflect the important role for AP activity in IgAN compared to other kidney diseases.

Sequencing of the genes for MBL and L-ficolin, *MBL2* and *FCN2*, identified association between future kidney failure and the *MBL2* variant rs1800450-A in IgAN. The at-risk allele was also associated with reduced MBL plasma levels and severe tubulointerstitial damage [49]. This indicates genetic variants can influence susceptibility to severe IgAN by determining low plasma MBL. Associations between LP genetic variants and IgAN have been identified in IgAN patient cohorts from China only. The reasons for this have not been established.

## Mechanisms of complement activation in IgA nephropathy

The development of effective complement-targeting therapeutic agents, and the safe selection of patients to receive these medications, is dependent on understanding the mechanisms that link complement activation and IgAN severity. In vitro analysis demonstrated that plate-bound, purified, human IgA was able to trigger C3 cleavage and the complement cascade via the AP but not CP [50]. This was unexpected because IgA is traditionally regarded as non-complement fixing. Notably, C3 activation was demonstrated only after treatment of the purified IgA with the cross-linking agent succinimidyl 3-(2-pyridyldithio)propionate [50]. C3 activation was greater with larger molecular weight aggregates. Supportive evidence of the pathogenicity of IgA polymers was provided by a rat model of IgA-mediated nephritis. Glomerular IgA and C3 deposition representative of human IgAN was dependent on the use of polymeric but not monomeric IgA [51].

Surface-bound IgA purified from pooled normal human serum can bind MBL-MASP complexes in vitro, leading to C3 and C4 activation and deposition [52]. Again, this is seen when microtitre plates are coated with polymeric, but not monomeric, IgA [52]. MBL binding to IgA was calcium dependent and could be blocked by pre-incubating IgA with specific saccharides. This suggested MBL-IgA interactions occurred between the carbohydrate recognition domain of MBL and specific glycosylation moieties on polymeric IgA. IgA fractions from individual IgAN patients reproduced strong MBL binding by polymeric, but not monomeric, IgA. However, large variability in MBL binding was seen between IgA from different patients [52]. Also, no difference in the quantification of MBL binding per unit of immobilised IgA was seen between patients and control samples [52]. Although these in vitro data provide important insight of complement activity in IgAN, a number of key questions remain unanswered. Firstly, why does glomerular IgA deposition cause significant AP activity, as opposed to the CP activation normally associated with IgA-IgG immune complexes? Secondly, why does AP activation seem to be dependent on polymeric but not monomeric IgA? Is this due to increased propensity to deposit in the glomerular mesangium, limited traffic through and disposal from the mesangium or increased complement activation on polymeric complexes, both in circulation and after glomerular deposition? Finally, how does the potential binding of MBL to IgA fit with the importance of galactose-deficient IgA1 to IgAN pathogenesis? Increased plasma levels of galactose-deficient IgA1, with exposed GalNac, are associated with IgAN. L-ficolin, but not MBL, is predicted to bind GalNac via exposed acetyl groups [53]. Therefore, the binding of MBL to IgA is likely more complicated than canonical PRM binding to exposed and pathogenic carbohydrate moieties on IgA1.

The analysis of other proteins potentially complexed to monomeric and polymeric IgA could help understand complement activity in IgAN. Proteomic assessment of circulating IgA1-containing immune complexes from patients has revealed the presence of C3 breakdown products including iC3b, C3c and C3dg [54, 55]. Therefore, the complement-activating ability of polymeric IgA could be explained by the presence and abundance of complexed complement proteins, which could vary markedly between individuals. The demonstration of AP activation on purified, circulating IgA1-containing immune complexes, which are the IgAN effector molecules, suggests that IgA immune complexes are complement-activating surfaces. In support of this, mesangial cell activation by immune complexes formed from cord blood galactose-deficient IgA1 and either anti-glycan IgG or patient-derived IgA1-specific IgG is dependent on a heat-sensitive serum factor, presumably complement [56].

The relevance and applicability of data from animal models of IgAN is limited by key differences in IgA. Only humans and hominoid primates have IgA1. Small animal IgA is similar to the human IgA2 subclass. IgA1, but not IgA2, is the dominant subclass in IgAN glomerular deposits. Nonetheless, animal models have provided insight into mechanisms, such as TP-dependent glomerular injury, that could contribute to IgAN pathogenesis. In a rat model of human mesangioproliferative glomerulonephritis, C5b9 induced immune stimulatory signals, such as the production of IL-6 and TGF-1β, and mesangial cell apoptosis that likely contribute to glomerular injury in IgAN [57, 58]. Using a Sendai virus model of IgAN, C5a receptor knockout mice had less proteinuria, reduced glomerular C3 and IgA deposition, and improved kidney histological changes compared with controls [59]. This was supported by the demonstration that human mesangial cell stimulation by IgA1 was reduced by C5aR antagonists [59].

Insight into mechanisms of complement dependent kidney injury has been provided by research into other kidney diseases. Similar to IgAN, a familial subtype of C3 glomerulopathy, CFHR5 nephropathy, is characterised by persistent microscopic haematuria, episodes of synpharyngitic macroscopic haematuria, recurrence in renal transplantation and more severe clinical course in males [13]. Patients with CFHR5 nephropathy have a heterozygous internal duplication of gene *CFHR5* exons 2 and 3. This leads to expression of a mutant protein with duplicated dimerisation domains. The mutant FHR5 has been shown to bind to C3b, iC3b and C3dg immobilised on a Biacore chip with greater affinity than native FHR5 [32]. Also, when added to a FH-dependent complement haemolysis assay, serum-derived mutant FHR protein preparations showed significantly greater haemolysis and complement deregulation than healthy controls [32]. The mechanism that causes kidney disease in CFHR5 nephropathy has recently been demonstrated in humanised mice that expresses the human mutant FHR5 and FH [60]. Mice that co-expressed human FH and mutant FHR5 developed glomerular C3 deposition [60]. Mice that expressed human FH and normal, wild-type human FHR5 did not. Glomerular C3 deposition in mutant FHR5 mice was reduced by delivering a FH molecule with surface C3 binding that was superior to native FH [60]. These data demonstrated C3 glomerulopathy can be caused by disruption of the homeostatic regulation of complement in the kidney. Similar mechanisms could contribute to glomerular inflammation and injury in diseases characterised by glomerular complement activation, such as IgAN.

## Clinical trials

Complement-mediated diseases, such as paroxysmal nocturnal haemoglobinuria, atypical haemolytic uraemic syndrome and C3 glomerulopathy, have led to the development of therapeutic inhibitors that target specific complement proteins [4]. Therapeutic complement inhibitors had been used to treat patients with IgAN on a case-by-case basis. Recently, trial participants with IgAN have been treated with investigational medicinal products that target lectin, alternative and terminal complement activation arms. Although many trials are ongoing with results awaited, some reported data is supportive of the pathogenic role of complement activation in IgAN.

Eculizumab is a humanised, recombinant, monoclonal antibody that inhibits C5 convertase activity and thereby prevents C5a release, TP activation and formation of C5b9. The treatment of IgAN with eculizumab has been reported in three case reports. Two of the three described transient improvement in clinical parameters when eculizumab was used for the treatment of crescentic IgAN that had progressed to significant kidney impairment despite the use of combination immunosuppression regimens [61, 62]. The third reported treatment of post-transplant recurrent, crescentic IgAN that had progressed to transplant failure [63]. In this case, eculizumab resulted in temporary stabilisation, but not improvement, of kidney function.

Avacopan (CCX168) is a small molecule inhibitor that targets the TP by binding the C5a receptor (C5aR). Avacopan limits the anaphylatoxin and pro-inflammatory effects of C5a but, unlike eculizumab, does not affect C5b9 formation. This may preserve innate immune and pathogen defence functions of the TP. The effect of avacopan on IgAN was studied in 7 patients in an open-label phase 2 study [64]. Proteinuria reduction, indicating clinical improvement, was achieved in 6 of 7 participants [64]. It will be important to test this result in larger, randomised, placebo-controlled studies.

Narsoplimab (OMS721) is a humanised monoclonal antibody to MASP-2 that inhibits LP activity. Following the initial observation that narsoplimab led to proteinuria reduction despite corticosteroid dose weaning in 4 patients with IgAN, the efficacy of 12 weekly intravenous narsoplimab infusions on reduction of proteinuria was tested. Twelve IgAN patients were randomised in a 1:1 ratio to narsoplimab or vehicle control [65]. All patients had persistent proteinuria of at least 1 g per 24 h despite maximal tolerated angiotensin-converting enzyme inhibitor (ACEi) or angiotensin receptor blocker (ARB) treatment, and eGFR of at least 30 ml/min/1.73 m^2^. Three patients were excluded for protocol violations. Reported adverse events were similar, and eGFR remained stable in the majority of patients in both cohorts. By week 18 (treatment plus 6 weeks’ follow-up), there was no difference in proteinuria reduction between the two cohorts (18.0% and 18.4%) [65]. All patients then received weekly infusions of narsoplimab for a further 12 weeks, with subsequent median cohort improvement in proteinuria of − 61.4%, although reduction was not seen in all patients (range change in proteinuria of + 7.3 to − 77.3%) [65]. Based on these results, a randomised, double-blind, placebo-controlled trial of narsoplimab is underway in IgAN patients with persistent proteinuria (ARTEMIS-IGAN, ClinicalTrials.gov identifier NCT03608033).

Results are pending from ongoing clinical trials of other complement inhibitors that target the AP and TP. These include a phase 2 and phase 3 trial of iptacopan (LNP023), an oral small molecular inhibitor of factor B (APPLAUSE-IgAN, ClinicalTrials.gov identifier NCT04578834); a phase 2 trial of pegcetacoplan (APL-2), a PEGylated compstatin that prevents C3 cleavage and activation (ClinicalTrials.gov Identifier NCT03453619); a phase 2 trial of ravulizumab, a long-acting, humanised monoclonal inhibitor of C5 activation (ClinicalTrials.gov identifier NCT04564339); and a phase 2, randomised, placebo-controlled study of cemdisiran, a small interfering RNA that is predicted to reduce liver production of C5 (ClinicalTrials.gov identifier NCT03841448). In addition to providing more treatment options for individuals with IgAN, data from these ongoing studies should provide further insight into the pathogenicity of complement in IgAN.

## Conclusions

A recent and growing body of research indicates that complement activity contributes to IgAN pathogenesis, most likely by influencing disease severity (Fig. 2). Although a number of key questions remain unaddressed, current data indicate genetic variants predispose patients to dysregulated and imbalanced complement activation, both on circulating IgA-containing immune complexes and in glomeruli, with subsequent glomerular inflammation and kidney injury. Variation in complement deposition could reflect the different contribution of the AP, LP and TP to kidney disease severity in each IgAN patient. Therefore, the demonstration of a single pathogenic mechanism for kidney injury in IgAN may be unrealistic. An effective future approach could be to describe the range of mechanisms through which complement activity can influence IgAN. This will identify pathogenic complement proteins, novel therapeutic targets and biomarkers for IgAN and other complement dependent kidney diseases.
